# Disassociated rhamphotheca of fossil bird *Confuciusornis* informs early beak reconstruction, stress regime, and developmental patterns

**DOI:** 10.1038/s42003-020-01252-1

**Published:** 2020-09-21

**Authors:** Case Vincent Miller, Michael Pittman, Thomas G. Kaye, Xiaoli Wang, Jen A. Bright, Xiaoting Zheng

**Affiliations:** 1 Vertebrate Palaeontology Laboratory, Division of Earth and Planetary Science, The University of Hong Kong, Pokfulam, Hong Kong SAR, China; 2 Foundation for Scientific Advancement, Sierra Vista, Arizona 85650 USA; 3 Institute of Geology and Paleontology, Linyi University, Linyi, 276000 Shandong China; 4 Department of Biological and Marine Sciences, University of Hull, Hull, HU6 7RX UK; 5 Shandong Tianyu Museum of Nature, Pingyi, 273300 Shandong China

**Keywords:** Palaeontology, Zoology, Biomechanics, Evolution, Evolutionary developmental biology

## Abstract

Soft tissue preservation in fossil birds provides a rare window into their anatomy, function, and development. Here, we present an exceptionally-preserved specimen of *Confuciusornis* which, through Laser-Stimulated Fluorescence imaging, is identified as preserving a disassociated rhamphotheca. Reconstruction of the in vivo position of the rhamphotheca validates the association of the rhamphotheca with two previous confuciusornithid specimens while calling that of a third specimen into question. The ease of dissociation is discussed and proposed with a fourth specimen alongside finite element analysis as evidence for preferential soft-food feeding. However, this proposition remains tentative until there is a better understanding of the functional role of beak attachment in living birds. Differences in post-rostral extent and possibly rhamphotheca curvature between confuciusornithids and modern birds hint at developmental differences between the two. Together, this information provides a wealth of new information regarding the nature of the beak outside crown Aves.

## Abstract (web summary)

Miller et al. present a disassociated rhamphotheca of a Cretaceous bird, which sheds light on the diet and developmental biology of some of the earliest birds. Exceptionally preserved fossils are rare but when soft tissue is preserved it can provide insight into the anatomy and behavior of extinct organisms.

## Introduction

**Fig. 1:**
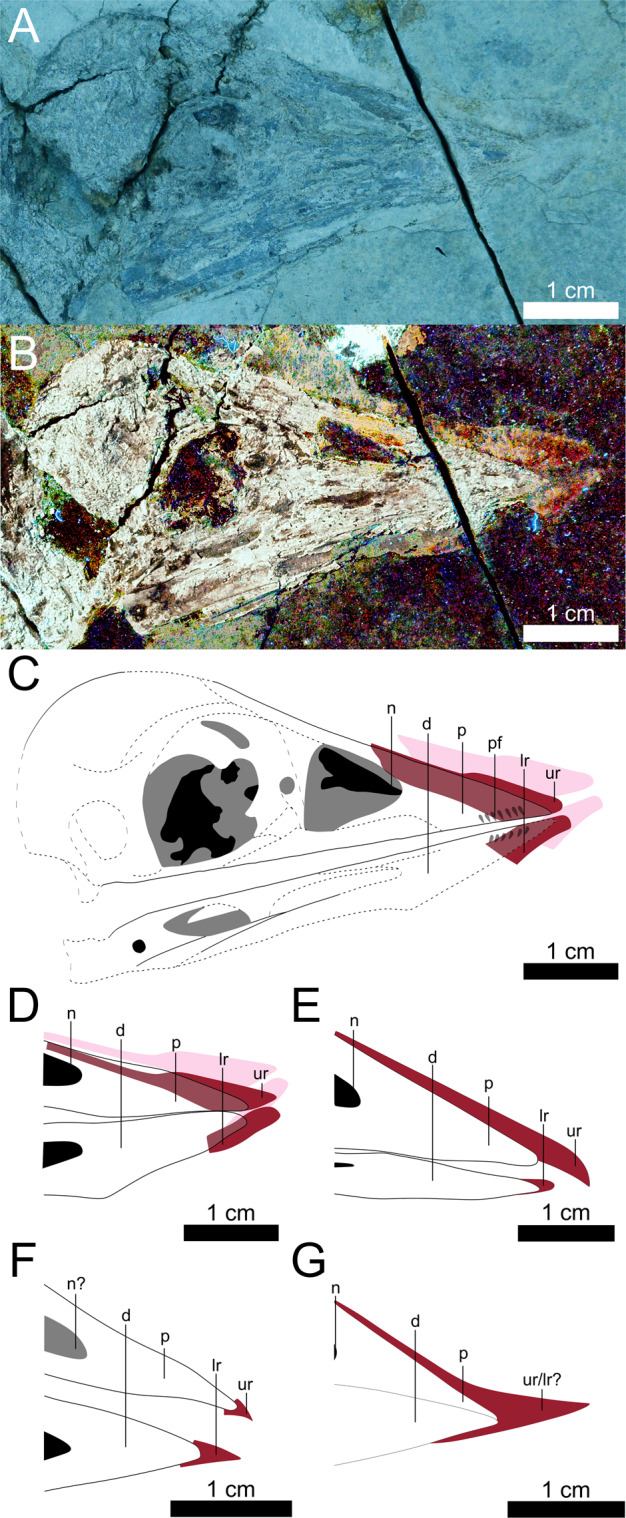
Comparison of confuciusornithid rhamphothecae. The skull of STM 13-162 (**a**) under white light and (**b**) under laser-stimulated fluorescence and photostacked with two images to improve clarity. The rhamphotheca is preserved as a brown stain near the premaxilla. The dorsal margins of the premaxilla and upper rhamphotheca are separated and subparallel. The upper rhamphotheca is more complete than the lower rhamphotheca and the rounding of both their tips appears to be genuine. The caudal extent of the upper rhamphotheca is obscured (see **c**). (**c**) Reconstruction of STM 13-162, with absent skeletal information (dotted lines and lower saturation) filled in from a previous reconstruction^3^. The pink shape is the in situ position of the rhamphotheca. The red shape is our reconstruction of the in vivo positioning of the rhamphotheca based on aligning the dorsal edge of the upper rhamphotheca with that of the premaxilla and then aligning the tips of the upper and lower rhamphothecae. We interpret the in situ upper rhamphotheca as extending just caudal to the prominent crack, to the point of color transition in the fluorescing area (mid-naris in vivo; anterior naris in situ). (**d**–**g**) Line drawings of the rostra and rhamphothecae of (**d**) BMNHC-PH986 (scale approximate based on STM 13-162, reconstructed by same methods), (**e**) IVPP V12352 (vertically reflected), (**f**) IVPP V11977 (vertically reflected), and (**g**) IVPP V11553 (vertically reflected). All are in lateral view except for the dentary of (**f**) which is in in ventral view. (**d**) Is redrawn from^6^ ^p. 156^, (**e**–**g**) are redrawn from^4^. d Dentary, lr lower rhamphotheca, n naris, p premaxilla, pf premaxillary foramina (vascularization), ur upper rhamphotheca.

Early-diverging, short-tailed confuciusornithids are the earliest birds (we use birds in reference to the Avialae) known to have fully edentulous keratinous beaks^1–4^, which displayed great disparity in form^1,4,5^. *Confuciusornis dui* had a curved beak^1^ whilst beaks were straight in *Confuciusornis sanctus*^4,6^ and *Eoconfuciusornis zhengi*^5^. Confuciusornithid beaks have been treated as structurally equivalent to those of modern birds^1,7,8^, but it is unclear to what extent these assumptions are valid given that beaks evolved convergently in modern birds (Aves) and non-avian avialans^9–12^. Here we show that such traditional assumptions should be more carefully applied in light of a new *Confuciusornis sanctus* specimen STM 13-162 in which laser-stimulated fluorescence (LSF) imaging reveals a disassociated straight-shaped keratinous beak (rhamphotheca) (Fig. 1). Firstly, we comment on uncertainties in the in vivo location of confuciusornithid keratinous beaks and the implications this has on the interpretation of reported specimens. Secondly, we propose that *Confuciusornis* exhibits loose beak attachment which, likened to some modern birds, may indicate that the beak operated in a low stress regime suited for feeding on softer foods. The latter exposes the importance of more rigorous study of beak attachment in modern birds. Finally, we comment on differences in the keratinous and bony beak anatomy between confuciusornithids and modern birds which points to subtle developmental differences whose origins would be a worthwhile target of future developmental studies.

## Results

### Taxonomy

STM 13-162 is referred to Confuciusornithidae based on its edentulous upper and lower jaws, prominent rostral and caudal dentary fenestrae, and pedal digit I ungual being smaller than those of other digits. It is more precisely referred to *Confuciusornis* based on its suboval fenestra perforating the deltopectoral crest (considered absent in *Eoconfuciusornis zhengi*) and pedal digit I being less than half the length of pedal digit II (proportionally shorter than in *Changchengornis hengdaoziensis* or *Yangavis confucii*)^13,14^. The two species of *Confuciusornis* considered valid, *C*. *sanctus* and *C*. *dui*, are differentiated by the dentary which is only partially preserved in STM 13-162. We believe that the bone visibly entering the more rostral dentary fenestra is the remnant of the triangular ventral process of the surangular, which is absent in *C. dui*^13^. Furthermore, the dorsal process of the maxilla in STM 13-162 is more cranially-positioned than in *C*. *dui*, consistent with *C*. *sanctus* (compare Fig. 1c, d in Wang et al.^13^). On these grounds we refer STM 13-162 to *Confuciusornis sanctus*.

### Description

In STM 13-162 the dorsal margins of the premaxilla and upper keratinous beak (rhamphotheca) are subparallel and separated (Fig. 1a, b). BMNHC-PH986 is an additional specimen of *C. sanctus* that has a disassociated rhamphotheca in a similar state of separation^6^ ^p. 156^, but has not been commented on before. The rhamphothecae of IVPP V12352^4^, IVPP V11977^4,5^, and IVPP V11553^1,4^ have no visible separation between the rhamphotheca and premaxilla. The rhamphotheca of STM 13-162 appears undeformed, as in BMNHC-PH986^6^ ^p. 156^, IVPP V12352^4^, and IVPP V11977^4,5^. The extent of deformation of the rhamphotheca of IVPP V11553 could not be evaluated due to loss of the original specimen^4^.

### Reconstruction

We reconstruct the in vivo position of the rhamphotheca in STM 13-162 in Fig. 1c by aligning the dorsal edge of the upper rhamphotheca with that of the premaxilla and then aligning the tip of the lower rhamphotheca with that of the upper one. The reconstructed rhamphotheca is straight and extends rostrally only a short distance past the premaxilla (Fig. 1c). The rostrocranial extent and dorsoventral thickness of the reconstructed rhamphotheca (Fig. 1c) is similar to the reconstructed rhamphotheca of BMNHC-PH986 (Fig. 1d) and the in situ rhamphothecae of IVPP V12352^4^ (Fig. 1e) and IVPP V11977^4,5^ (Fig. 1f). The rhamphotheca of IVPP V11553^1,4^ (Fig. 1g), which may or may not be in situ, projects farther rostrally and is more curved than in STM 13-162, BMNHC-PH986, IVPP V12352^4^, and IVPP V11977^4,5^. All specimens except IVPP V12352 have a rhamphotheca that project caudally to around the level of the rostrocranial midpoint of the naris, without encircling it (Fig. 1c, d, f, g,^4^). It is unclear if termination of the rhamphotheca rostral to the naris (Fig. 1e) represents the in vivo state in IVPP V12352 or if the rhamphotheca is simply incomplete.

### Finite element modeling

**Fig. 2:**
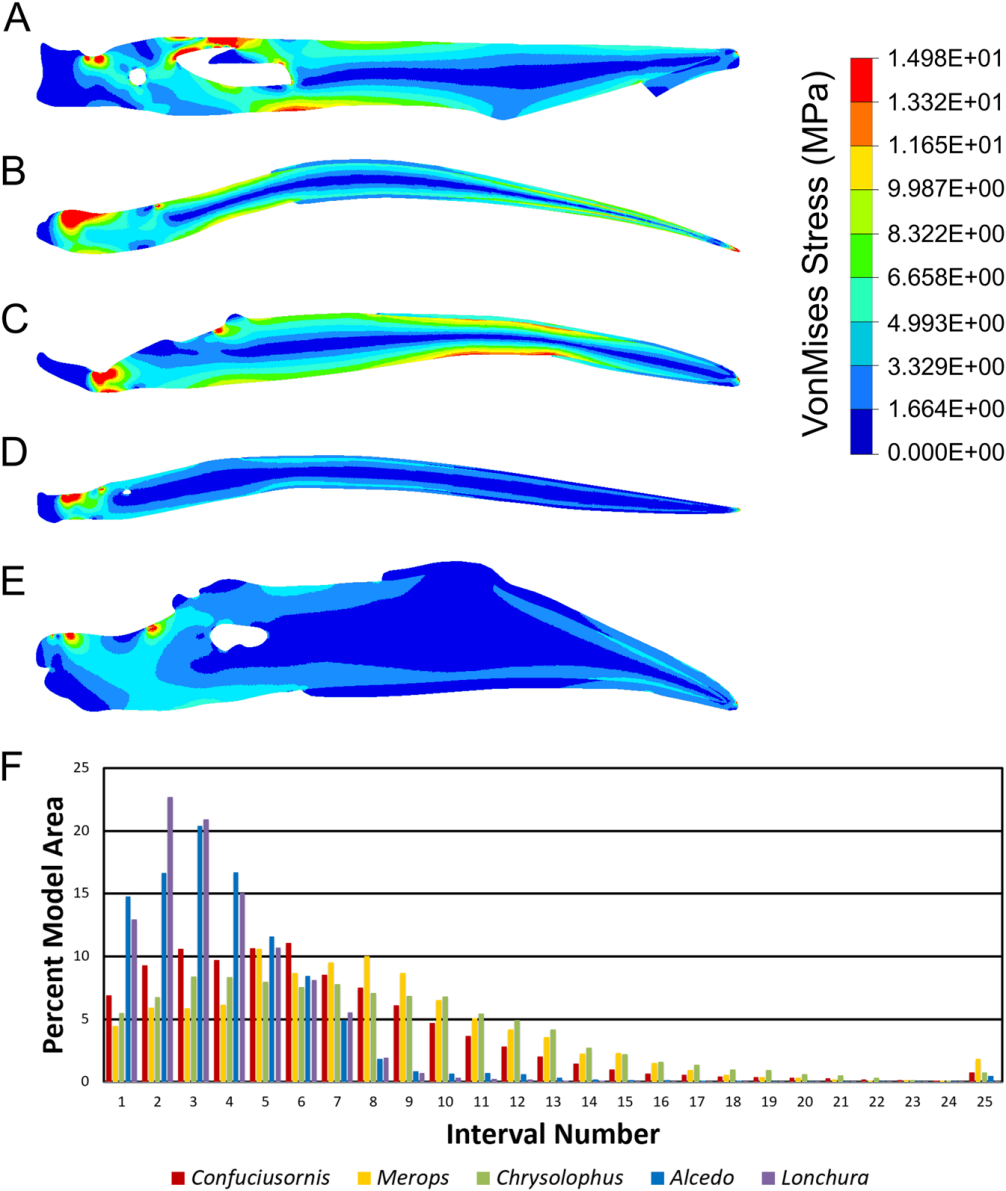
*Confuciusornis sanctus* STM 13-162 compared to extant sally-striking and granivorous birds. The mandible as reconstructed in Fig. 1c is converted into a two-dimensional Finite Element model (**a**) and compared to models of a sally-striking bee-eater, *Merops orientalis* (**b**), an herbivorous pheasant, *Chrysolophus pictus* (**c**), an aquatic-gleaning kingfisher, *Alcedo atthis* (**d**), and a granivorous finch, *Lonchura malacca* (**e**). The mandible of *C. sanctus* reacts to loading like a sally-striker or herbivore, with areas of high stress (warm colors) more similar in size and extent to that of *M*. *orientalis* and *C*. *pictus* than *A*. *atthis* or *L. malacca*. Results of the intervals method for comparing finite element models^47^ (**f**) corroborate this interpretation. Bar height indicates the percent area of a model which experiences a given interval of stress, with higher interval numbers indicating higher Von Mises stress. The majority of the model area in *L. malacca* and *A*. *atthis* is under low amounts of stress. Model area of *C*. *sanctus*, *M. orientalis*, and *C*. *pictus* show similar trends in stress throughout, with a positively-skewed distribution peaking at intervals of moderate stress.

Finite element modeling of the lower jaw of *C*. *sanctus* (Fig. 2) returns a stress regime more comparable to our chosen representatives for sally-strikers and herbivorous birds than to those for gleaning predators or seed crushers.

## Discussion

The disassociation of the keratinous beak (rhamphotheca) in STM 13-162 challenges the implicit assumption that existing preserved confuciusornithid rhamphothecae maintain their original orientation in life^1,4,5^. The subparallel and separated dorsal margins of the premaxilla and upper rhamphotheca in STM 13-162 and BMNHC-PH986 have the same spatial relationship as keratinous beaks in extant avian skeletal specimens that dislocated rostrally post-mortem (CV Miller, personal observation). This suggests that a similar dislocation occurred post-mortem in STM 13-162. STM 13-162 exhibits a degree of crushing comparable to other confuciusornithid specimens^3,4,6^, suggesting that similar dislocations of the horny beak cannot be ruled out in other specimens.

The in vivo position of the rhamphotheca in STM 13-162 (Fig. 1c) allows validation of the in vivo placement of two previously-reported confuciusornithid rhamphothecae. The similar rostrocranial extent and dorsoventral thickness of the rhamphotheca of the reconstructions of STM 13-162 (Fig. 1c) and BMNHC-PH986 (Fig. 1d) to IVPP V12352^4^ (Fig. 1e) and IVPP V11977^4,5^ (Fig. 1f) suggests that the in situ position of the latter two rhamphothecae represent the in vivo state. The rhamphotheca of IVPP V11553^1,4^ (Fig. 1g) projects further rostrally and is more curved compared to STM 13-162, BMNHC-PH986, IVPP V12352, and IVPP V11977 (Fig. 1c–f). This suggests that the rhamphotheca in IVPP V11553 has been taphonomically altered or represents a different in vivo morphology from other confuciusornithids.

The rhamphotheca of STM 13-162 provides important data about how the rhamphotheca was structured in confuciusornithids. In modern birds, the beak dermis is anchored to the underlying bone via collagen fibres (Sharpey’s fibres)^15,16^. Similar structures also anchor the cornified palatal epithelium of turtles^17^. This form of anchoring is present in the phylogenetic bracket of beaked reptiles and is widespread in vertebrates^18^, so should be present in confuciusornithids. While it is possible that any collagen fibres may have decayed away after death, and that STM 13-162 and BMNHC-PH986^6^ ^p. 156^ represent a more advanced stage of decay than other specimens, decay studies of modern birds show the skull detaching from the body before any parts of the skull (including the rhamphotheca) separate from one another^19,20^. The combination of an articulated skull and postcrania but a disassociated rhamphotheca in both STM 13-162 and BMNHC-PH986 would seem to imply that the rhamphotheca of *Confuciusornis* was relatively loosely attached in life. In finches, Sharpey’s fibres tend to be more numerous in areas of higher stress^15^. This suggests that the strength of beak attachment might be a proxy for in vivo stress, although the functional implications of modern bird beak attachments are still not well understood. In this context, the beak of *Confuciusornis* may have experienced a relatively lower stress regime in life, but this will require further testing as larger fossil and living bird datasets become available. Despite its disassociation, the shape of the rhamphotheca itself does not appear to be deformed in either STM 13-162 or BMNHC-PH986 (Fig. 1b,^6^ ^p. 156^), suggesting that it was not particularly soft or malleable (*contra*^8^).

Confirmation of a low stress regime can provide insight into the palaeoecology of *Confuciusornis*. Extant animals that consume softer foods tend to have jaws that are less well adapted to the high stress regimes associated with processing hard foods^21–24^. While our preliminary finite element models cannot currently distinguish between sally-striking and generalized herbivory, they do indicate that the stress regime in the *Confuciusornis* mandible is incongruent with gleaning predation and oral processing of hard foods (Fig. 2). Claims that it had a strong lower jaw^8^ ^p. 446^ are also unsubstantiated. We therefore propose that *Confuciusornis* and other confuciusornithids would have preferentially taken soft foods, or that any granivory involved pecking and then processing in the gizzard, rather than oral processing (although gastroliths and seed remains seen in other Mesozoic birds^7,25^ are unknown in confuciusornithids, suggesting that this is unlikely). These conclusions are contingent on the taxa selected being adequate representatives of their feeding style. Future tests with larger sample sizes in each category should allow for more reliable and precise characterization of feeding in *Confuciusornis*. Our FEA findings are further supported by a cranial mechanical advantage calculated at 0.117 and a straight beak (Supplementary Fig. 1), which falls within the range of grabbing/gleaning, pecking/grazing, and probing uses of beak during feeding described by Fig. 5 in Navalón et al.^26^. While these claims are tentative and vulnerable to taphonomic bias, we wish to highlight that the functional implications of beak attachment are worth exploring in further detail.

Comparisons between the keratinous and bony beak anatomy of confuciusornithids and modern birds provide an opportunity to better understand rhamphotheca formation in early birds. In modern birds, rhamphotheca formation is still poorly understood at a molecular level^12,27^. Recent studies have investigated gross morphological trends in rhamphotheca form relative to the underlying bone^27–29^, which presumably stems from common developmental systems. Rostrocaudal extent of the rhamphotheca, relative to homologous regions of the skull, is one of the most predictable rhamphotheca characteristics in modern birds. The caudal extension of the rhamphotheca in *Confuciusornis* to the approximate level of the rostrocranial midpoint of the naris, without encircling it (Fig. 1c, d, e, g,^4^), resembles all but a few later-diverging avian taxa^28,29^ ^p. 34,^ ^30^. However, the relatively short rostral projection of the rhamphotheca in confuciusornithids despite their highly vascular rostra (Fig. 1c–f,^4,5^) is in stark contrast to modern birds. In avians, the rhamphotheca tends to extend farther rostrally beyond the skull with increased vascularization of the rostrum (i.e., larger premaxillary foramina)^29^ ^p. 35^. As opposed to the other confuciusornithid specimens, IVPP V11553^1,4^ has a longer rostral projection of the rhamphotheca as expected in modern birds, but a shape unlike modern birds. Rhamphotheca shape is controlled by more complex developmental factors than rostrocaudal extent^12^ and is in turn more difficult to quantify^29,31^. The simplest metric of shape is the difference in curvature between the rhamphotheca and the premaxilla. In modern birds, the rhamphotheca is generally more curved than the premaxilla. As the premaxilla becomes more curved, the curvature of the rhamphotheca increases disproportionately^29^ ^p. 58,^ ^31^. STM 13-162 (Fig. 1), IVPP V12352^4^, and IVPP V11977^4,5^ all follow this trend. IVPP V11553^1,4^ runs contrary to this trend in having a straight premaxilla and curved rhamphotheca more similar to some tortoises^30^. Confuciusornithids display rhamphotheca traits similar to modern birds in some ways (caudal extent, shape in most) and completely contrary in others (rostral projection, shape in IVPP V11553). This supports the hypothesis that similar mutations across the Dinosauria triggered the initial formation of beaks (possibly mediated by BMP4^11^) before differing minutiae in their development (e.g., differing activation of proliferation centres^27^) led to the diversity of forms seen in the dinosaur fossil record^11,12^.

In summary, modern imaging techniques have revealed heretofore unknown detail of the rhamphothecae of early fossil birds. Assumptions regarding the in vivo position and thickness of the rhamphotheca should be made with caution as disassociation of the rhamphotheca and beak is possible, though from our reconstruction two specimens^4,5^ appear to not have undergone disassociation. This potential for disassociation is tentatively suggested as an indicator of low in vivo jaw stress regimes and an impetus for further study of beak attachment in modern birds. Finite element modeling based on the new reconstruction also suggests a weak jaw. Signs of differential development between confuciusornithids and later-diverging birds include the disconnect between premaxillary foramina and rostral extent of the rhamphotheca as well as the potential mismatch between premaxilla and rhamphotheca curvature. Our characterization of the beak condition in confuciusornithids provides crucial new insights into beak structure and development outside of crown Aves. Future specimens will be particularly valuable in developing increasingly better-supported reconstructions of edentulous fossil species, potentially elucidating biomechanical implications of beak detachment, and deepening understanding of the pathways and mechanisms of rhamphotheca formation.

## Methods

LSF imaging was performed according to an updated version of the methodology of^32,33^ so only a brief description of the method is provided here. A 405 nm laser diode was used to fluoresce the specimen following standard laser safety protocol. Thirty second time exposed images were taken with a Nikon D810 DSLR camera and 425 nm blocking filter. Image post-processing (equalization, saturation, and color balance) was performed uniformly across the entire field of view in *Photoshop CS6*. Figure 1b was photostacked in this program to maximize the clarity of the skull and rhamphotheca in the final image.

Reconstruction of the skull and rhamphotheca were made in *CorelDRAW X8*. Reconstruction of the skull in Fig. 1c, d is based primarily on an overlay of an existing skull reconstruction from Fig. 4 of Elzanowski et al.^3^ onto an image of STM 13-162 (see caption of Fig. 1); this reconstruction was used in all subsequent analyses.

Mechanical advantage was examined using the methods of Navalón et al.^26^: the inlever is treated as the distance from the articular condyle to the centre line of the jaw adductor muscles (locations approximated based on those of avians as mapped by Holliday^34^) and the outlever as the distance from the articular condyle to the rostral tip of the premaxilla (disregarding the rhamphotheca). Measurements were taken on a scaled image in *CorelDRAW X8* using the ‘Parallel Dimension’ tool.

Previous studies have proposed various dietary modes for *Confuciusornis*, including sally-striking^3,8,35^, generalized herbivory^1,36^, surface gleaning from water^8,35^, and granivory^1,7^. We compared the mandible of *Confuciusornis* with those of extant birds representing each of these feeding behaviors using finite element analysis (FEA)^37^. FEA comparisons are based on the principles set forth by Dumont et al.^21^, in which a structure is relatively weaker if it experiences higher peak Von Mises (VM) stress under the same relative load. Analysis was restricted to two dimensions as STM 13-162 is only preserved two-dimensionally, and so relative model loadings and plane strain criteria were chosen based on the suggestions of Marcé-Nogué et al.^38^. Scaled forces were applied using the muscle simulation method of Morales-García et al.^39^ to recreate the *m. adductor mandibulae externus*, mapped onto the bone after Holliday^34^. All models used homogeneous, isotropic material properties for the skull and rhamphotheca found to produce results similar to in vitro strain gauge data in ostriches by Cuff et al.^40^ (*E* = 7000 MPa, *ν* = 0.35 for bone, *E* = 3000 MPa, *ν* = 0.35 for rhamphotheca). Models were constrained from translation in all axes at the articular glenoid, and dorsoventrally at the rostral tip of the rhamphotheca. Mandibles were used for comparison to eliminate confounding factors associated with multiple non-feeding functions and cranial kinesis in the avian upper jaw. Mandibles of *Merops orientalis* (the green bee-eater, a sally-striker), *Chrysolophus pictus* (the golden pheasant, a herbivorous galliform), *Alcedo atthis* (the common kingfisher, an aquatic surface-gleaner), and *Lonchura malacca* (the tricolored munia, a granivorous estrildid finch) were taken from Skullsite^41–44^. Rhamphotheca thickness is not visible from external pictures and varies greatly between birds (compare^45,46^). Sensitivity analysis (Supplementary Table 1) found VM stress to be insensitive to moderate changes in rhamphotheca thickness, so extant species models were built to be consistent with the model of *Confuciusornis* (dorsoventral thickness ~20% rhamphotheca and 80% bone). The intervals method^47^ was used to compare VM stress among the models, with trends converging at 25 intervals. Twenty-five equal intervals were constructed based on the highest stress experienced in any model, 14.98 MPa (disregarding the top 2% of values as singularities per the recommendation of^47^), making each interval 0.5992 MPa. All models were created and solved within HyperWorks 2019 Student Edition (*HyperMesh* and *Optistruct*, Altair Engineering Inc.).

### Reporting summary

Further information on research design is available in the Nature Research Reporting Summary linked to this article.

## Supplementary information

Supplementary Information

Reporting Summary

## Data Availability

The specimen used in this study, STM 13-162, was collected in Toudaoyingzi, Jianchang, Liaoning Province, China from rocks belonging to the Aptian Jiufotang Formation. It is currently housed at the Shandong Tianyu Museum of Nature, Linyi, Shandong, China. Models created in this study and a spreadsheet of their inputs and outputs are available on Mendeley: 10.17632/mpzjpf7xfk.2.

## Code availability

R code for performing the intervals method of finite element model comparison is available in the Supplementary Information of Marcé-Nogué et al.^47^.
