# Supplementary material for: Disassociated rhamphotheca of fossil bird Confuciusornis informs early beak reconstruction, stress regime, and developmental patterns
Source: Commun Biol. 2020 Sep 21;3:519. doi: 10.1038/s42003-020-01252-1 (PMC7506531; doi:10.1038/s42003-020-01252-1)
Supplement: Supplementary file 1 — Supplementary Information [file 42003_2020_1252_MOESM1_ESM.docx]

**Supplementary Information**


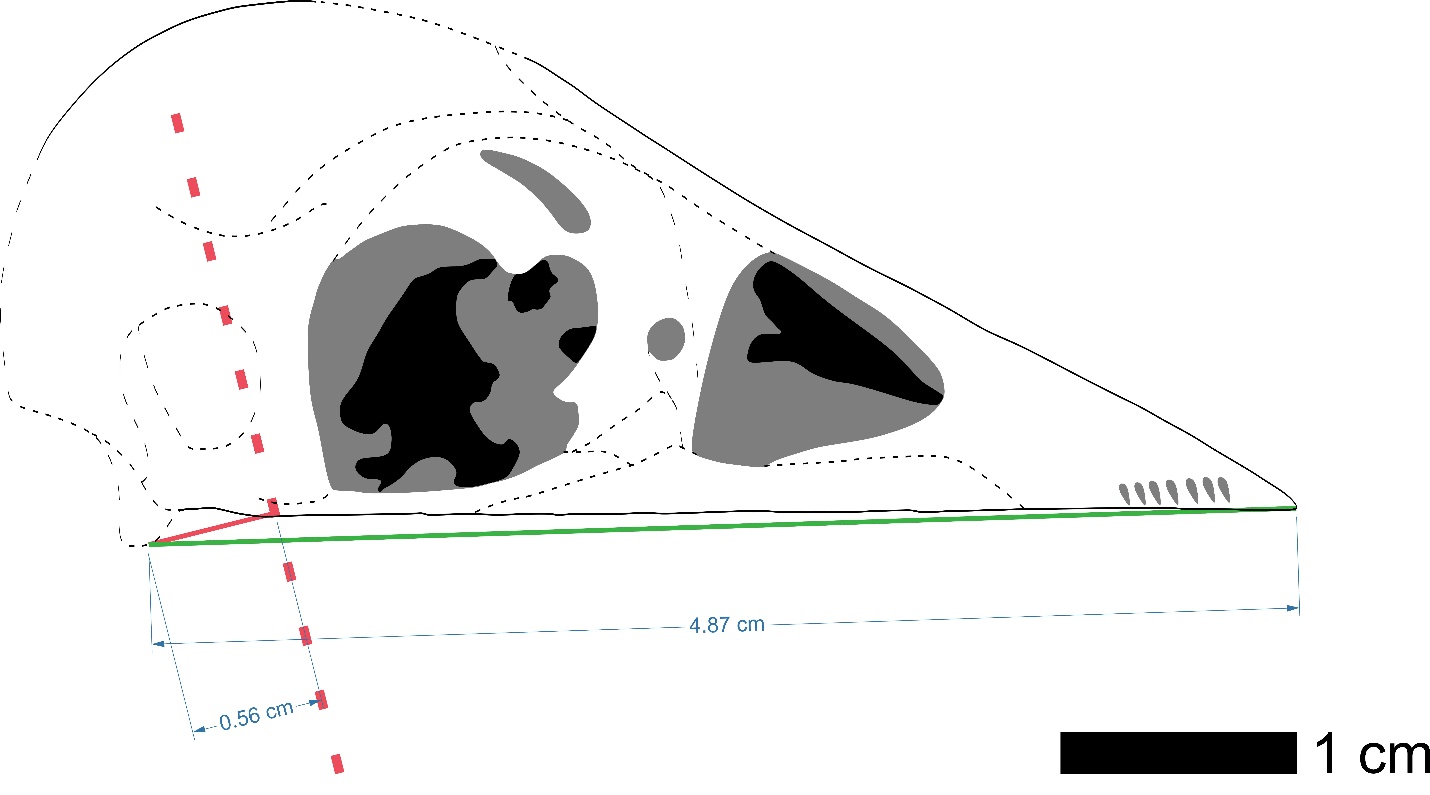


**Supplementary Figure 1. Reconstruction of *Confuciusornis* *sanctus* STM 13-162.** The maxilla as reconstructed in Figure 1C, with rhamphotheca removed. Mechanical inlever (red) and outlever (green) are labeled after^1^, with lengths labeled.

| **Rhamphotheca Thickness** | **Max VM Stress (MPa)** | **Avg VM Stress (MPa)** |
| --- | --- | --- |
| 0% | 5.609 | 2.19662 |
| 25% | 5.827 | 2.24624 |
| 50% | 5.695 | 2.25848 |
| 75% | 5.839 | 2.25643 |
| 100% | 5.891 | 2.28961 |
| 125% | 5.507 | 2.1151 |
| 150% | 5.864 | 2.24471 |
| 175% | 6.081 | 2.32951 |
| 200% | 6.102 | 2.30009 |

**Supplementary Table 1. Sample Sensitivity Analysis of Rhamphotheca Thickness Effect on FEA.** Example based on the model of *Lonchura malacca* (Fig. 2E) in which sensitivity was relatively high in order to present a conservative estimate. VM stress was worked with disregarding the upper 2% of values as singularities after^2^. Rhamphotheca thickness is given relative to that used in Figure 2. Thickness was modulated by increasing or decreasing the amount of underlying bone, so as to not affect the overall lever length of the jaw. While there is a weak trend of increase (R^2^ = 0.4) in the maximum VM stress experienced with increased thickness, likely due to compressing the tip of the mandible to smaller elements, the overall average VM stress resembles pure noise (R^2^ = 0.1). The range of VM stress within a taxa (0.6 MPa) is also much less than the range between taxa (8.9 MPa).

**Supplementary References**

1. Navalón G, Bright JA, Marugán-Lobón J, Rayfield EJ. The evolutionary relationship among beak shape, mechanical advantage, and feeding ecology in modern birds. *Evolution* **73**, 422-435 (2018).

2. Marcé-Nogué J, De Esteban-Trivigno S, Püschel TA, Fortuny J. The intervals method: a new approach to analyse finite element outputs using multivariate statistics. *PeerJ* **5**, e3793 (2017).
